# A transcriptomic dataset of supplemental red-light effects on solid-stem development in two spring wheat varieties

**DOI:** 10.1016/j.dib.2026.113281

**Published:** 2026-09-23

**Authors:** Lavanya Mendu, Nagendra Pratap Singh, Akshara Bathini, Venugopal Mendu

**Affiliations:** a USDA, Agriculture Research Service, Crop Genetics Research Unit, 141 Experiment Station Rd, Stoneville, MS, 38776, USA; b Department of Agriculture, Agribusiness, and Environmental Sciences, Texas A&M University, Kingsville, TX, 78363, USA; c Department of Plant Sciences and Plant Pathology, Montana State University, Bozeman, MT, 59718, USA

**Keywords:** Wheat stem sawfly resistance, Phytochrome signalling, Red-to-far-red ratio, Pith parenchyma, RNA-seq, Programmed cell death, Stem solidness scoring

## Abstract

Wheat stem sawfly (WSS) (Cephus cinctus Norton) is a major pest of hard red spring wheat, causing significant yield losses. Genetic resistance conferred by solid stem wheat genotypes remains the most effective strategy for mitigating WSS damage. However, pith development, a key determinant of stem solidness and resistance, exhibits considerable variation among genotypes and is strongly influenced by environmental factors, particularly light quality. This study addresses the gap in understanding how light regulated molecular pathways contribute to stable and uniform pith expression. To elucidate these mechanisms, two contrasting spring wheat varieties, Duclair (solid-stemmed) and Reeder (hollow-stemmed), were grown under standard greenhouse lighting (GL) and supplemental red-light (SL) treatments. We performed comprehensive transcriptomic profiling to identify light-responsive and genotype-specific changes in gene expression. Differential expression, Gene Ontology, and KEGG enrichment analyses revealed that supplemental red light significantly modulates transcriptional pathways associated with phytochrome signalling, pith parenchyma differentiation, secondary cell wall modification, and programmed cell death processes tightly linked to pith formation. These findings provide a molecular framework for understanding how enhanced red light promotes uniform pith development in solid-stemmed wheat. By clarifying the biological pathways underlying light-modulated stem solidity, this dataset supports breeding and biotechnological strategies to improve resistance to the wheat stem sawfly. This dataset identified approximately 12,041 differentially expressed genes, with 8007 upregulated, and 4034 downregulated in Duclair SL, compared to Reeder SL. Strengthening this resistance has direct implications for reducing lodging, yield loss, and economic damage associated with WSS infestation.

## Graphical abstract

**Figure:**
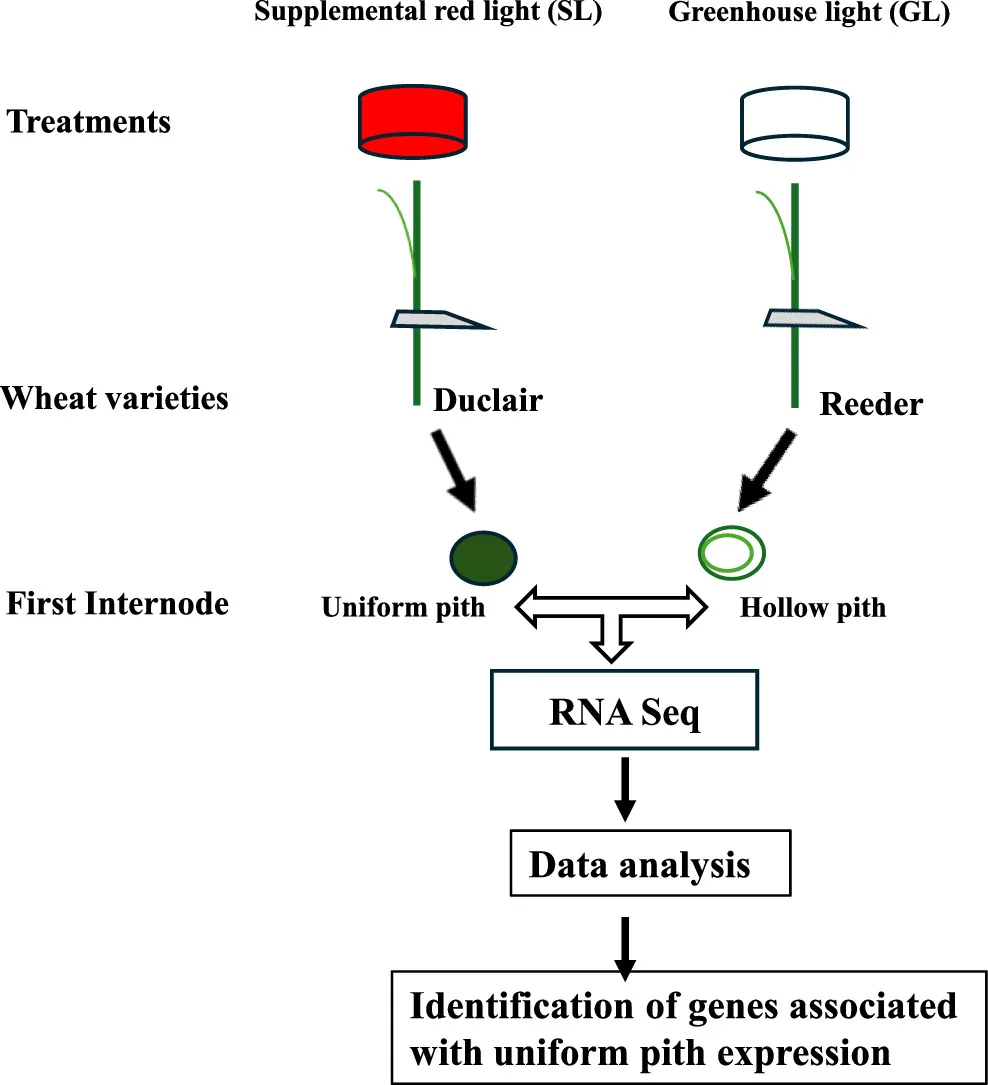
Visual representation of graphical abstract.

Specifications Table

**Table:** 

Subject	Biology
Specific subject area	Light regulated pith formation; wheat.
Type of data	Table, Analyzed, Processed Raw: FASTQ files (paired-end RNA-seq reads) Processed; DEG lists (CSV); GO and KEGG enrichment results (CSV) Image (PNG)
Data collection	First internode tissue samples are collected from spring wheat varieties, Duclair and Reeder, grown under both supplemental red light (SL) and standard greenhouse light conditions (GL) [1].
Data source location	The dataset includes two varieties, Duclair and Reeder.
Data accessibility	Repository name: NCBI Sequence Read Archive (SRA) Data identification number: Bio Project PRJNA1462305 (SRA submission SUB15571597) Direct URL: https://www.ncbi.nlm.nih.gov/bioproject/PRJNA1462305 DEGs and GO Data files https://doi.org/10.6084/m9.figshare.32273610
Related research article	Yes(Mendu, Bathini et al. 2026)

## 1 Value of the Data

• Supplemental red light provides a powerful tool for modulating pith development, particularly in spring wheat varieties where solid stem is a critical trait for resistance against wheat stem sawfly (WSS).
• The transcriptomic dataset generated under supplemental red-light conditions reveal that red light significantly alters gene expression profiles associated with phytochrome signaling, pith parenchyma differentiation, secondary cell wall modification, and programmed cell death, all processes associated with uniform pith formation.
• The gene regulatory network provided by RNA seq analysis under supplemental red light provided insights into biological underpinnings of light modulated solid stem trait that support breeding and biotechnological strategies aimed at improving yield, reducing lodging, and minimizing economic losses due to WSS infestation.
• Application of supplemental red light is strongly justified as it enables targeted manipulation of developmental and defense pathways, offering a robust foundation for improving spring wheat insect resistance and productivity.

## 2 Background

Wheat stem sawfly (WSS) is a major constraint in spring wheat production, causing substantial yield losses across major growing regions [1], The most effective and widely adopted management strategy is genetic resistance derived from solid stem varieties [2] where pith-filled stems reduce oviposition, larval development and larval survival in the stems, thereby limiting WSS infestation [3,4]. However, the expression of stem solidness and consequently the degree of WSS resistance varies considerably among genotypes and is strongly influenced by environmental conditions, particularly light quality and photoperiod [[5], [6], [7]]. Previous studies have demonstrated that resistant bread wheat varieties exhibit variable resistance to WSS, with light intensity being a critical environmental factor. Reports indicate that moderate shading or a 50% reduction in light intensity can slightly decreases stem solidness and WSS resistance, while severe shading leads to hollow stems and markedly increased susceptibility [8]. These findings underscore the positive impact of higher light intensity on solid stem development: High light intensity enhance pith formation, whereas shaded light reduces pith expression and increases vulnerability to WSS [9]. Although these environmentally driven phenotypic shifts are well documented, the molecular mechanisms linking light perception to parenchyma development remain poorly understood. Prior research has suggested that genotype by environment interactions play a central role in determining the consistency of solid stem expression [10].

Yet, the light-regulated transcriptional pathways underlying this trait have not been comprehensively characterized. This study addresses this knowledge gap by integrating pith phenotypes with transcriptomic profiles from contrasting spring wheat genotypes grown under supplemental red-light (SL) and standard greenhouse conditions (GL). The resulting dataset provides new molecular insights into the gene networks that govern light-modulated pith differentiation, offering a foundation for improving the stability of stem solidness and enhancing WSS resistance in spring wheat.

## 3 Data Description

This dataset offers a comprehensive transcriptomic resource for investigating how supplemental red light (SL) influences gene expression during early stem elongation (Zadok 31) stage in spring wheat. First internode tissue samples were collected from two contrasting genotypes, Duclair (solid stemmed) and Reeder (hollow stemmed), cultivated under SL and GL conditions. The experimental design comprised of 12 RNA seq libraries (2 genotypes X 2 light treatments X 3 biological replicates). Raw paired-end reads were quality filtered using fastp (v0.21.0) [11] and high-quality (HQ) reads were aligned to the *Triticum aestivum* IWGSC CS RefSeq v2.1 genome using STAR (v2.7.11b) [12]. Across all twelve libraries, at least 96.9% of reads were retained as high quality with unique mapping rates ranging from 84.87% to 89.57% (Table 1). Sequence output, quality metrics, and SRA accession numbers for each sample are summarized in Table 1. The raw FASTQ files are publicly available in the NCBI Sequence Read Archive under BioProject PRJNA1462305.

**Table 1:** RNA-seq sequencing output and alignment summary for all 12 samples.

Sample	Raw reads (bp)	HQ reads (bp)	HQ (%)	Unique map (%)	SRA accession
Duclair_GL_1	8687,433,300	8419,174,076	96.9	88.22	SRR38388169
Duclair_GL_2	10,343,683,200	10,059,325,078	97.3	86.71	SRR38388168
Duclair_GL_3	8883,936,900	8628,563,630	97.1	86.01	SRR38388167
Duclair_SL_1	10,586,551,200	10,258,841,538	96.9	84.87	SRR38388166
Duclair_SL_2	8454,990,600	8208,399,098	97.1	87.82	SRR38388165
Duclair_SL_3	9670,309,800	9377,407,166	97.0	86.97	SRR38388164
Reeder_GL_1	9200,203,200	8936,312,436	97.1	89.17	SRR38388163
Reeder_GL_2	9922,477,080	9637,407,488	97.1	89.57	SRR38388162
Reeder_GL_3	9544,470,600	9260,509,102	97.0	88.49	SRR38388161
Reeder_SL_1	10,090,937,700	9775,835,034	96.9	88.91	SRR38388160
Reeder_SL_2	9323,781,600	9030,711,494	96.9	86.15	SRR38388159
Reeder_SL_3	9357,781,200	9080,836,250	97.0	87.82	SRR38388158

Gene level counts were generated by RSEM (v1.3.3) and imported into DESeq2 (v1.38.0) for pairwise differential expression analysis across four contrasts: (1) Duclair SL vs. Duclair GL; (2) Reeder SL vs. Reeder GL; (3) Duclair GL vs. Reeder GL; and (4) Duclair SL vs. Reeder SL. Differentially expressed genes (DEGs) were defined as those with |log₂FC| ≥ 1 and an adjusted p-value ≤ 0.05, corresponding to a minimum two-fold change in expression. These analyses identified 12,576, 6265, 22,932, and 14,224 DEGs for the respective comparisons, with a core set of 168 DEGs shared across all four comparisons (Table 2). Complete DEG lists (CSV), along with Gene Ontology (Biological Process, Cellular Component, Molecular Function) and KEGG pathway enrichment outputs generated using clusterProfiler (v4.6.0). are provided as supplementary files at https://doi.org/10.6084/m9.figshare.32273610. Overall, this dataset captures light regulated and genotype specific transcriptional responses associated with phytochrome signaling, pith parenchyma differentiation, secondary cell wall modification, and programmed cell death processes mechanistically linked to uniform pith development under supplemented red-light conditions.

**Table 2:** Summary of differentially expressed genes (DEGs) and associated output files for each pairwise comparison.

Comparison	DEGs	Up	Down
Duclair SL vs. Duclair GL	12,576	7852	4724
Reeder SL vs. Reeder GL	6265	2146	4119
Duclair GL vs. Reeder GL	22,932	12,925	10,007
Duclair SL vs. Reeder SL	14,224	8843	5381

## 4 Experimental Design, Materials and Methods

### 4.1 Plant material, growing conditions and light treatments

Two spring wheat varieties, Duclair and Reeder were cultivated at the Montana State University, Plant Growth Center (Bozeman, MT (45.6669° N, 111.0561° W). Seeds were individually sown in 9-inch plastic pots containing pre-moistened Sunshine Mix #1. Plants were maintained under controlled environmental conditions: 22 °C during the day, 18 °C at night, and a 16-hour light/ 8-hour dark photoperiod until reaching Zadoks growth stage 31.

Light Treatments:

Greenhouse Light (GL): Plants received a combination of natural greenhouse light and supplemental illumination from Philips SON AGRO 430 W high-pressure sodium lamps (Philips, Somerset, NJ, USA) for 16 h day light. The combined spectrum encompassed the photosynthetically active radiation (PAR) range (400 to 700 nm), with an increased proportion of blue light relative to standard greenhouse lighting. The spectroradiometer graph showed a wavelength peak ranging between 400 and 700 nm with integrated total for photon flux density as 188.6 µmol·m^−2^·s^−1^nm^−1^ during daytime. This suggests 40 W/m^2^ (watts of light energy per square meter) during daytime.

Supplemental Red Light (SL): Plants were exposed to natural greenhouse light supplemented with Lumi Grow PRO 325 narrow-spectrum LED fixtures (Emeryville, CA, USA) for 16 h per day. These LEDs provided distinct spectral wavelengths: red (601 to 700 nm), blue (400 to 500 nm), and white light (501 to 600 nm), thereby enhancing the PAR range. In the daytime the spectral output by spectroradiometer readings was characterized by a pronounced peak within the 600 to 700 nm range, aligning with the red region of the PAR spectrum. The integrated total for photon flux density of 344.6 µmol·m^−2^·s^−1^nm^−1^suggests 63.83 W/m^2^ during daytime.

### 4.2 Tissue collection for RNA sequencing

Tissue samples were collected from the first internode of Duclair and Reeder plants at Zadoks growth stage 31. To accurately identify this stage, the developing internode was located above the soil surface visually to confirm its position. Then, the plants were transferred to lab, collected stems and using a sterile blade a -transverse cut was made at the center of the identified internode of each stem. Then, transferred on to a slide for observing microscope, while the flanking internode tissue on either side of the section was immediately excised, flash frozen in liquid nitrogen, and stored at −80 °C. The sectioned stem tissue was examined microscopically to verify pith structure and ensure consistency with later developmental observations at Zadoks stage 49. Three independent biological replicates were collected per variety and treatment (2 varieties × 2 treatments x 3 replicates = 12 samples). All samples were shipped on dry ice to the sequencing facility (IDseq). where RNA extraction, quality assessment, library preparation, and sequencing were performed as illustrated in Fig. 1.

**Fig. 1:**
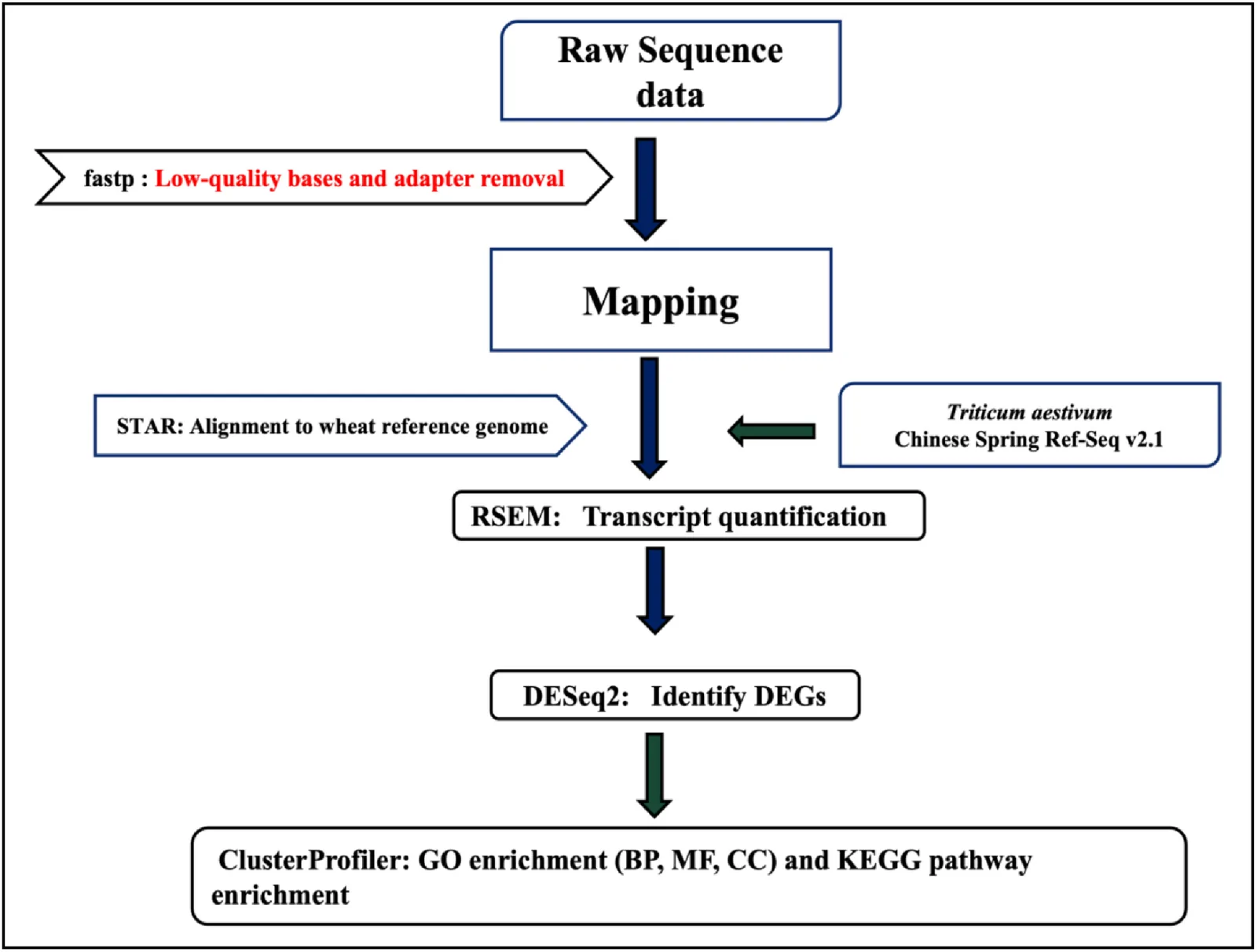
RNA‑sequencing analysis pipeline.

### 4.3 RNA extraction, quality assessment, library preparation and sequencing

Total RNA was extracted from each internode sample, and RNA integrity was evaluated using RNA Integrity Number (RIN) values. Samples with RIN values of at least 7.5 were retained for downstream library preparation. Poly(A)⁺ mRNA was enriched from 1 µg of total RNA per sample, chemically fragmented, and reverse-transcribed to generate first-strand cDNA. second-strand synthesis, end repair, dA-tailing, adapter ligation, and PCR amplification. The final library showed a mean fragment size of 430 bp corresponding to an insert size of 300 bp. Libraries were combined at equimolar concentrations and sequenced on the DNBseq T7 platform using 150 bp paired-end mode, targeting approximately 40 million read pairs per sample.

### 4.4 Quality control, adapter trimming, and quantification

Raw FASTQ files were processed using fastp (v0.21.0) [12] (Table 1) for quality filtering, with automatic adapter detection and a minimum per-base quality score of Q30. High-quality reads were aligned to the *Triticum aestivum* IWGSC CS RefSeq v2.1 reference genome using STAR (v2.7.11b) [12] employing key non-default parameters: to optimize mapping and quantification: -outSAMtype BAM SortedByCoordinate; –outSAMattributes NH HI AS NM; –quantMode TranscriptomeSAM GeneCounts; and–outFilterMultimapNmax 1 to retain only uniquely mapping reads. The genome index was built using–genomeSAindexNbases 14, appropriate for the ∼17 Gb hexaploid wheat genome. Gene-level expected counts were estimated from STAR-aligned BAM files using RSEM (v1.3.3) [13].

### 4.5 Differential expression analysis

Gene-level count matrices were imported into R (v4.2.0) and analyzed with DESeq2 (v1.38.0) for differential expression analysis [14]. Genes with total counts <10 across all 12 samples were removed. Size factors were estimated using the default median-of-ratios method and pairwise contrasts tests were evaluated using the Wald test; Differentially expressed genes (DEGs) were defined using thresholds of |log₂FC| ≥ 1 (equal to two-fold gene expression) and Benjamini-Hochberg adjusted p-value ≤ 0.05. Four contrasts combinations were computed: (1) Duclair SL vs. Duclair GL; (2) Reeder SL vs. Reeder GL; (3) Duclair GL vs. Reeder GL; (4) Duclair SL vs. Reeder SL (Table 2). To identify the common and differential transcriptional responses, overlap among the four DEG sets was visualized and measured using the Venn Diagram R package (v1.7.3).

### 4.6 Functional enrichment analysis

A T. aestivum specific annotation database was generated with makeOrgPackageFromNCBI() function in AnnotationForge (v1.40.0) (R/Bioconductor), incorporating NCBI RefSeq annotations for taxon ID 4565. Gene Ontology (GO) enrichment for Biological Process (BP), Cellular Component (CC), and Molecular Function (MF) categories was performed using enrichGO () in clusterProfiler (v4.6.0) [15], with a Benjamini-Hochberg-adjusted p-value cutoff of 0.05 and a minimum gene set size of 10. KEGG pathway enrichment was executed using enrichKEGG() with the organism code ‘taes’ (*T. aestivum*) and the same statistical *p*-value threshold. All enrichment analyses were performed separately for up and down-regulated genes within each set of the four pairwise comparisons. Table 2 summarizes the results.

## Limitations

▪ This dataset evaluates two spring wheat genotypes. Incorporating multiple solid stem varieties with diverse genetic backgrounds will help in further validating the consistency of light induced transcriptional changes associated with uniform pith development.
▪ This dataset focuses on a single developmental stage (Zadoks 31). Although this stage is critical for pith parenchyma differentiation, additional sampling across earlier and later developmental windows would provide a more comprehensive view of the temporal dynamics governing pith formation and wheat stem sawfly resistance.

## Ethics Statement

Authors have read and follow the ethical requirements for publication in Data in Brief and confirming that the current work does not involve human subjects, animal experiments, or any data collected from social media platforms.

## CRediT Author Statement

The current study is conceived by **VM and LM**. Internode tissue samples were collected by **AB, LM, and NPS**. Samples were collected and shipped by **AB, NPS, and LM**. First draft was written by **NPS, LM, and VM**. Revisions are made by **NPS, LM, and VM**. Transcriptomics sequencing data analysis by **NPS**. Data interpretation **NPS, LM, and VM**. Draft writing and revisions: **NPS, LM, and VM**.

## Disclaimers

USDA is an equal opportunity provider, employer, and lender.

Mention of trade names or commercial products in this publication is solely for the purpose of providing specific information and does not imply recommendation or endorsement by the U.S. Department of Agriculture. The findings and conclusions in this manuscript are those of the author(s) and should not be construed to represent any official USDA or U.S. Government determination or policy.

## Data Availability

SRABio Project PRJNA1462305 (SRA submission SUB15571597) (Original data).
